# Generative Artificial Intelligence Terminology: A Primer for Clinicians and Medical Researchers

**DOI:** 10.7759/cureus.49890

**Published:** 2023-12-04

**Authors:** Oleksiy Melnyk, Ahmed Ismail, Nima S Ghorashi, Mary Heekin, Ramin Javan

**Affiliations:** 1 Department of Radiology, George Washington University School of Medicine and Health Sciences, Washington D.C., USA

**Keywords:** generative artificial intelligence, convoluted neural network, deep learning (dl), large multimodal model, large language model, natural language processing, artificial intelligence, chatgpt, gpt-4

## Abstract

Generative artificial intelligence (AI) is rapidly transforming the medical field, as advanced tools powered by large language models (LLMs) make their way into clinical practice, research, and education. Chatbots, which can generate human-like responses, have gained attention for their potential applications. Therefore, familiarity with LLMs and other promising generative AI tools is crucial to harness their potential safely and effectively. As these AI-based technologies continue to evolve, medical professionals must develop a strong understanding of AI terminologies and concepts, particularly generative AI, to effectively tackle real-world challenges and create solutions. This knowledge will enable healthcare professionals to utilize AI-driven innovations for improved patient care and increased productivity in the future. In this brief technical report, we explore 20 of the most relevant terminology associated with the underlying technology behind LLMs and generative AI as they relate to the medical field and provide some examples of how these topics relate to healthcare applications to help in their understanding.

## Introduction

At the one-year anniversary of the public release of ChatGPT and with the rapid growth of generative artificial intelligence (AI) and large language models (LLMs), it is important for all healthcare professionals to develop an understanding of the related basic concepts and terminology [1]. This familiarity can expand the contribution of a wide range of individual talents, interests, and capabilities joining research teams in the medical field, potentially advancing healthcare as a whole and across each specialty. While these concepts are not new in the informatics world, given the explosion and resurgence of interest, a revisit is warranted, especially for individuals unfamiliar with the technical aspects of these concepts and their utility and implementation in healthcare. Our hope is to make the definitions understandable for anyone in the medical field without prior knowledge of these topics, allowing individuals to approach the topic without hesitation and at a deeper level.

For physicians, knowledge of how these tools function allows for more informed adoption of AI assistance in diagnosis, treatment planning, and patient monitoring. For medical researchers, AI methodologies such as deep learning (DL) and natural language processing (NLP) are enabling complex analysis of large datasets to uncover novel insights and accelerate discoveries. An appreciation of the nature and limitations of AI can help clinicians evaluate the appropriateness of these tools for patient care and researchers to direct innovation and fill knowledge gaps. Overall, literacy in fundamental AI concepts empowers healthcare professionals and researchers to actively shape the responsible integration of AI in medicine rather than passively accept externally driven applications, especially by the commercial sector. This knowledge helps realize these transformative technologies’ potential to expand clinical capabilities and enhance patient outcomes.

## Technical report

**Figure 1:**
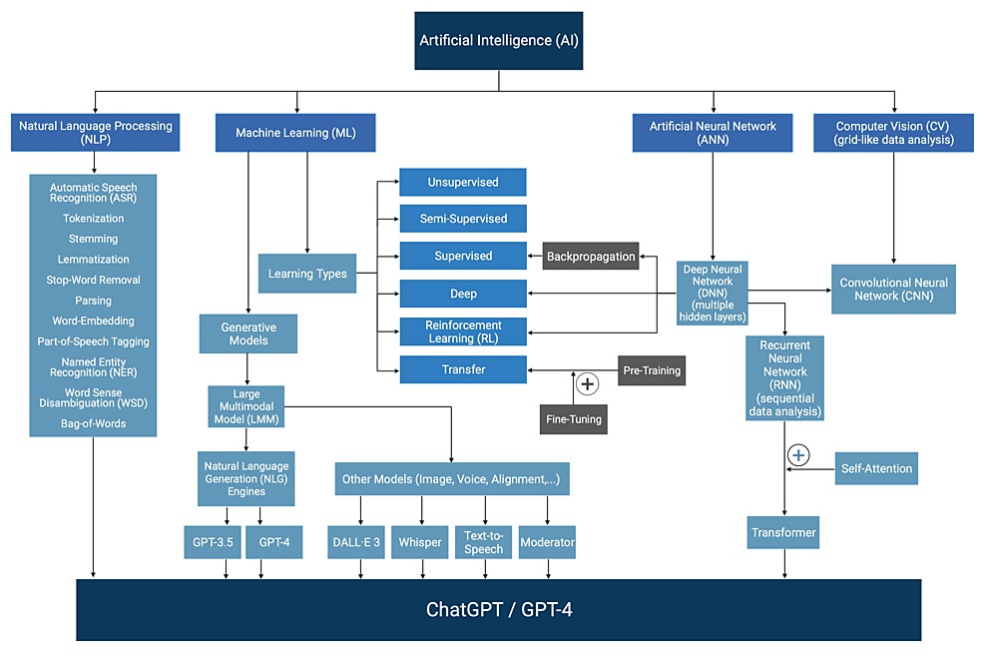
A mind map of large language model and generative artificial intelligence terminology.

We outline 20 terms pertaining to the understanding of LLMs and generative AI in a succinct, structured, and progressive manner and show their interrelations. A detailed mind map is further provided to enhance comprehension of these terms and their connections (Figure 1).

Artificial intelligence

AI refers to computer programs capable of performing tasks mimicking human intelligence using algorithms and statistical models to process information and make decisions, often in real-time [2]. Generative AI refers to the creation of various types of content, including text, images, audio, video, and synthetic data. Artificial general intelligence (AGI) is when AI reaches a point where it possesses human-like abilities to learn, adapt to new problems, and apply generalized learned knowledge to other contexts similar to an average human [3].

Machine learning

Machine learning (ML) is a branch of AI that enables computer programs to learn from data by identifying patterns and improving performance through experience. ML often requires large datasets and benefits from iterative feedback and fine-tuning [4].

Artificial neural networks

Artificial neural networks (ANNs) are algorithms with an architecture inspired by the human brain and biological neural networks. ANNs consist of interconnected nodes, or artificial “neurons,” forming an input layer, hidden layers for processing information, and an output layer [2,5]. Neural networks can learn from examples of relationships in data and are capable of complex pattern recognition and decision-making.

Deep learning

DL is a subfield of ML that uses deep neural networks (DNNs) for data analysis, pattern recognition, and decision-making, with the distinct capability of autonomously learning crucial features for predictions with minimal human intervention [6]. DNNs are multiple layers of weighted, interconnected nodes trained through supervised or unsupervised learning [2]. The term “deep” refers to the number of layers within the neural network, ranging from several to thousands. DNNs can extract features from raw data using a learning algorithm called “backpropagation” [7]. As a result, they have the capacity to learn from mistakes and adjust their course over time to solve problems. DNNs are more powerful than traditional ML algorithms but require large amounts of data and computational resources.

Supervised versus unsupervised learning

Supervised learning is a form of ML where models are trained on labeled data to make predictions, with the data quality influencing model performance. On the other hand, unsupervised learning involves models identifying patterns and structures in unlabeled data, such as clustering similar data points or finding concise representations, without any prior examples or feedback [2,8].

Backpropagation

Backpropagation is a supervised learning technique used to adjust the connection weights of a neural network to minimize the error between its predicted and actual output. It is used in DNNs during the training phase to adjust weights and optimize network performance [7,8]. This is done by computing the error between predicted and actual outputs, propagating it backward through the layers, and adjusting the weights [7,8]. The process is typically repeated over many iterations, or “epochs,” until the error is sufficiently minimized.

Reinforcement learning

Reinforcement learning (RL) is a type of unsupervised learning where the model learns to make decisions by receiving feedback as rewards or penalties in a problem-oriented environment [2]. The goal is to find the optimal sequence of actions that maximizes results. RL uniquely maximizes reward signals instead of finding hidden structures like traditional ML models [9]. Reinforcement learning from human feedback (RLHF) incorporates human feedback during the training process, known as “alignment” [10].

Natural language processing

NLP is a field of AI that analyzes and processes free text into structured language data [5]. NLP tasks include, but are not limited to, translation, semantic analysis, automatic summarization, question-answering, and speech recognition [2,11,12]. For familiarity with some of the important technical terminology in NLP see Table 1 [11].

**Table 1:** Natural language processing (NLP) terminology.

Term			Description	Application
Analysis	Text representation	Word embedding	A technique used to represent words in a multidimensional space, where semantically similar words are located closer to each other. It allows for semantic relationships to be captured between words and can be used to train machine learning models	To extract similar words from a body of text. For example, using word embeddings, a radiology report containing “lung nodule” can be linked to similar terms such as “pulmonary nodule” and “solitary pulmonary nodule”
	Syntax	Part-of-speech tagging	A process of labeling each word in a sentence with its corresponding part of speech (e.g., noun, verb, adjective). This is useful for understanding the structure of a sentence and can be used for tasks such as text classification and sentiment analysis	To extract relevant information such as the type of imaging study (e.g., CT, MRI) and the location of the abnormality (e.g., left lung, right kidney). For example, part-of-speech tagging can be used to identify that “MRI” is a noun and “right” and “left” are adjectives referring to the location of the abnormality
		Parsing	Parsing is the process of analyzing sentences to determine their grammatical structure. It involves breaking down a sentence into its components and identifying the relationships between these components	To extract relevant information, such as the indication for the study, the findings, and the impression. For example, a parser can identify the relationship between a finding and its location in the body, or between a finding and a modifier such as “new” or “improved.” This can help radiologists and other clinicians quickly identify important information in the report
	Semantics	Named entity recognition (NER)	Named NER is the process of identifying and categorizing named entities in text, such as people, places, and organizations	To extract information about patients, such as their name, age, and gender, as well as information about the imaging study, such as the modality, body part, and contrast agent used. For example, an NER system can extract the patient’s name from a radiology report and use it to link the report to the patient’s electronic health record. It can also extract the imaging study’s modality and body part to help with quality assurance and billing
		Word-sense disambiguation	The process of identifying the correct meaning of a word with multiple meanings in context	To differentiate between the different senses of words such as “mass.” In radiology reports, “mass” can refer to a neoplasm, a hematoma, or a benign mass, and this process helps identify the correct meaning of “mass” in the context of the report
	Morphological	Word stemming	The process of reducing words to their base or root form to simplify analysis. It removes prefixes and suffixes from words to reduce them to their core meaning	To recognize that the words “nodules” and “nodule” have the same root word, “nodul,” and groups them in the analysis to simplify analysis and identify patterns in large datasets
Non-analysis		Lemmatization	Similar to word stemming, lemmatization reduces words to their base form, but it uses a more sophisticated algorithm to do so. This process considers the context and its lexical knowledge base to get the correct base forms of words	To analyze large datasets of radiology reports by grouping related terms. Used to identify the base form of each word in the reports, such as “tumor” instead of “tumors.” This would allow the researchers to more accurately identify the frequency of specific conditions and treatments and to compare their results with other studies
	Bag of words		A simple technique to represent a document as a bag of words ignoring grammar and word order. Each word is assigned a weight that reflects its frequency or importance in the document	To identify and extract relevant medical concepts. It involves identifying the occurrence of words or phrases in the text and assigning them a “weight” based on their frequency or importance. For example, the Bag of Words model can be used to identify and extract terms such as “lung,” “opacity,” “nodule,” “mass,” etc., to help with diagnosis and decision-making
	Automatic speech recognition		Transcription of spoken language into text using machine learning techniques	To transcribe radiologist dictations, allowing the radiologist to more efficiently create a report. This can improve report turnaround times and patient care
	Tokenization		Breaking down a sentence or phrase into discrete units or tokens. These tokens represent linguistic units that can be strung together to represent more complex data	To break down a radiology report into individual words or phrases for analysis. This can aid in the identification of trends or patterns within the report or dataset, such as the frequency of certain diagnoses or the use of certain imaging modalities
	Stop word removal		The process of removing commonly used words that carry little to no meaning from a text	To remove words such as “the,” “a,” and “an” from radiology reports to focus on the keywords and concepts, reducing the size of the data being analyzed and improving processing speed

Autonomous agents

Autonomous agents are systems that can perform tasks and make decisions independently without human intervention. They perceive their surroundings through sensors, process them, reason about them, and take action to achieve their goals [2].

Recurrent neural networks

Recurrent neural networks (RNNs) are networks designed to process sequential data with temporal dependencies, such as time series or natural language, by utilizing internal loops that allow information to persist, essentially enabling a “memory.” This enables the networks to make decisions based on the input sequence and previous context rather than just each input independently. The recurrent connections allow RNNs to develop complex temporal representations critical for sequence modeling tasks such as language translation, speech recognition, and time series forecasting. However, RNNs often struggle with capturing long-distance dependencies, meaning they may have difficulty relating information from earlier steps in the sequence when the gaps are too large [5,13].

Self-attention

Self-attention is an iterative mechanism that enables models to assign varying degrees of importance to different parts of the input during processing, which is particularly useful in handling variable-length inputs and focusing on relevant information for making predictions. Computing attention score allows it to dynamically focus on the most relevant weights to produce more accurate predictions. Self-attention has been widely used in NLP tasks such as language translation and sentiment analysis, where the relationships between words in a sentence are important. Combined with RNN-like structures, it forms the backbone of transformer models, which are the central component of chatbot technologies [14,15].

Transformer

Transformer is a type of RNN architecture that employs self-attention, allowing it to focus on different parts of the input sequence simultaneously. This enables transformers to efficiently capture intricate relationships in input data and leverage parallelization, a technique that divides tasks into smaller subtasks executed concurrently across multiple processing units. This is particularly effective for NLP tasks involving lengthy sequences of speech/text [16,17].

Computer vision

Computer vision (CV) is AI that uses visual data inputs to analyze and interpret images/videos [2]. It can be applied for optical character recognition, image segmentation, image generation, object detection, and image-based search engines [2,12].

Convolutional neural networks

Convolutional neural networks (CNNs) are a type of DNN specialized for image data. They employ a hierarchical structure of layers to process images in sections, extracting features from nearby regions through convolutional layers to ultimately make predictions [5]. They are used for image classification, object detection, and image segmentation.

Pre-training

A technique in unsupervised DL where a model is trained on general unlabeled data before undergoing training for a specific task [18]. This gives the model a strong initial representation of the data, allowing for faster and better convergence during fine-tuning [15,18].

Fine-tuning

A technique that calibrates pre-trained models on smaller task-specific datasets to improve performance, commonly used in NLP and CV [2,15].

Transfer learning

A technique where a model pre-trained on one task is used for a new but related task to improve performance and reduce data and computational resource requirements [8]. In the context of transfer learning, transformers have been used as the pre-trained model, which is then fine-tuned for a specific NLP task. Models such as generative pre-trained transformer 4 (GPT-4) have been pre-trained on large amounts of text data and then fine-tuned for tasks such as sentiment analysis or named entity recognition with a small amount of task-specific data [19].

Generative models

Generative models are a type of ML model capable of learning the underlying probability distribution of data and are trained to generate similar, new unseen examples [20].

Language models

A type of generative model that learns to produce new text from text data [2,15]. Natural language generation (NLG) creates text similar to human-written text [20]. LLMs are neural network-based models with billions of text-based parameters [2]. Similar techniques are used in large multimodal models (LMMs), which can also incorporate additional inputs such as images or audio [3].

Generative pre-trained transformer 4

GPT-4 is the most well-known LLM with NLG capability, trained on over 1.75 trillion parameters [1]. LLMs utilize transformers to process long text sequences using self-attention and RNNs.

To provide clarity and avoid confusion, note that ANNs are basic foundational neural networks while DNNs are more complex with multiple hidden layers. Two subtypes of DNNs are RNNs and CNNs. RNNs handle sequential data and remember past inputs through loops, while CNNs are specialized for image data and detect spatial patterns through grid-like data analysis. Additionally, note that DL requires large amounts of labeled or unlabeled data to make predictions or decisions based on features of the data for tasks such as classification and pattern recognition. RL, on the other hand, does not require labeled data; instead, an agent interacts with the environment through trial and error to maximize rewards and achieve its goals. Additionally, as backpropagation, self-attention, and transformers are unique key concepts responsible for the development of LLMs and the latest rise of AI to the forefront of medical research, we take a deeper dive into their understanding.

Backpropagation is key to training DNNs to learn from mistakes through an iterative process of forward and backward passes. In the forward pass, input data is fed through the network and predictions are generated. The difference between the predicted and actual outputs is calculated as an error or loss function. The error value is propagated backward by attributing portions of the error to each neuron by calculating the gradient of the loss function. As the error flows backward, the connection weights between neurons are incrementally updated to reduce error. The learning rate controls the size of weight updates. Multiple rounds of forward and backward passes are performed until network accuracy is maximized. Understanding how error attribution and weight adjustments enable neural networks to “learn” from mistakes is key to comprehending the power of backpropagation.

Self-attention identifies the most relevant inputs by calculating attention scores between each pair of inputs. Inputs can attend to one another irrespective of their position, allowing modeling of long-range dependencies. Input words are first mapped to key, value, and query vector representations capturing semantic importance. Dot products are then taken between the query and key vectors to obtain attention scores reflecting the relevance of each input word. These attention scores are used to weigh each value vector in a summation, such that the output represents the most pertinent information for prediction. This selective focus attuned to input importance allows more accurate processing of long sequences such as sentences.

Transformers introduced a novel architecture that could handle longer sequences for tasks such as translation more effectively than RNNs and CNNs. It is characterized by an encoder-decoder structure, where the encoder maps an input sequence into a latent representation, and the decoder then generates the target sequence from this encoder output. Instead of processing inputs sequentially, transformers’ attention mechanism allows the model to identify relevant relationships regardless of distance. Another key feature is the integration of residual connections, where outputs are added to inputs at each layer, allowing information to flow across the entire network and improving optimization. As positional data is lost with self-attention, position encodings are added to input embeddings to retain order. Lastly, the transformer architecture inherently makes it conducive to parallel processing. The segmented modules and repetitive structure allow massively parallel computation, significantly reducing the overall training time of the model.

## Discussion

This overview of core generative AI and LLM concepts opens the door for individuals in healthcare with little familiarity with these technologies, potentially unlocking their tremendous transformative potential for reimagining every facet of healthcare. Key takeaways include: first, AI methodologies such as ML and neural networks enable the detection of complex data patterns to inform clinical decision-making and medical discoveries; second, NLP and CV facilitate analysis of text, images, and other qualitative data for optimized workflows and patient engagement; Third, RL provides a framework for iterative improvement of targeted tasks. Fourth, self-attention in combination with RNNs form transformers which are the core revolutionary technology behind chatbots. An in-depth discussion of the numerous use case applications and opinion papers written over the past year on generative AI is beyond the scope and purpose of this technical paper, with over 1,850 PubMed publications related to ChatGPT or GPT-4 alone as of the time of this submission. To provide a more tangible understanding of the key topics, examples of healthcare applications for key AI/ML concepts are provided in Table 2.

**Table 2:** Example healthcare applications of artificial intelligence/machine learning terminology.

Artificial intelligence terminology	Example healthcare applications
Machine learning	• Predicting patient outcomes and trajectories using large electronic health record (HER) datasets • Identifying personalized medication regimens based on pharmacogenomic data • Optimizing hospital logistics such as bed assignments and operating room schedules • Powering clinical decision support systems with updated recommendations
Computer vision	• Automated analysis of retinal images to detect diabetic retinopathy/macular degeneration • Interpretation of digital pathology slides to identify cancerous tissue • Tracking of Alzheimer’s disease progression through structural MRI changes • Detection of pneumonia, tuberculosis, and other thoracic diseases from chest X-rays • Monitoring of hand hygiene practices and personal protective equipment use through video feeds
Natural language processing	• Sentiment analysis of patient feedback to identify care experience improvements • Extraction of social determinants of health data from clinical notes • Automated coding of medical procedures from clinical documentation • Chatbots to provide medication and treatment reminders to patients • Voice-enabled virtual assistants for patient education and symptom checking
Reinforcement learning	• Optimizing sepsis care and ventilator settings for critically ill patients • Improving chemotherapy regimens through iterative learning • Personalizing glucose control for diabetes patients • Optimizing stroke rehabilitation strategies based on patient response
Deep learning	• Identifying disease-associated genetic variants from genomic datasets • Drug discovery through analyzing molecular target binding affinities • Predicting post-discharge outcomes using EHR data • Generating synthetic patient data for research with guaranteed privacy
Supervised learning	• Classifying skin lesions as benign or malignant using labeled dermatology images • Predicting 30-day hospital readmissions using discharged patient data/readmit labels • Predicting sepsis onset from vital sign time series data with sepsis labels • Detecting arrhythmias from labeled ECG time series waveforms • Diagnosing psychiatric disorders from questionnaires and psychological evaluations
Unsupervised learning	• Discovering new disease subtypes by clustering genetic or biomarker data • Identifying gaps in care protocols by finding anomalies in process data • Segmenting patient populations to target preventive interventions • Detecting healthcare fraud by identifying unusual usage patterns • Grouping patients by test results to identify clusters with similar lab value patterns

Significant challenges remain in developing and deploying generative AI in healthcare responsibly. A major limitation is that model performance is highly dependent on training data quality. Furthermore, available datasets may suffer from biases. Algorithms commonly lack full transparency, making it difficult to understand the rationale behind AI predictions and recommendations, a phenomenon known as the “black box” effect. Moreover, integrating AI into fast-paced clinical workflows remains challenging. Approving generative AI tools for clinical use faces regulatory hurdles. Moreover, questions remain regarding legal liability if AI errs in practice. Thoughtful solutions are needed to address these issues of reliability, accountability, and safety before AI can be responsibly adopted for sensitive tasks such as diagnosis and treatment planning in the clinical realm. More open discourse and conservative gradual utilization are warranted as generative AI continues to evolve. Issues of privacy, consent, equity, and accessibility must be proactively addressed. More interdisciplinary research and regulatory guidance focused on the highest standards of patient care are needed to achieve the full potential of generative AI in medicine.

## Conclusions

For healthcare professionals and medical researchers, consciously building AI literacy starting with core concepts is the first step to active engagement. This could involve evaluating tools with AI capabilities being implemented in one’s workplace, seeking continuing education opportunities, and monitoring developments through reputable sources. A mindset open to supplementing expertise with data-driven insights while identifying areas of persistent need for human judgment will enable clinicians to guide appropriate generative AI adoption. Clinicians and researchers should look for intersections within their specialty where LLMs and generative AI can accelerate discoveries or strengthen investigations. These technologies offer vast opportunities to enhance healthcare, improve efficiency, and reduce cost; however, realizing the benefits and mitigating the risks requires proactive partnerships across all parties involved, who should ideally be able to communicate effectively at a basic technical level.
